# Supplementary material for: Gastro-Esophageal Cancer: Can Radiomic Parameters from Baseline 18F-FDG-PET/CT Predict the Development of Distant Metastatic Disease?
Source: Diagnostics (Basel). 2024 Jun 6;14(11):1205. doi: 10.3390/diagnostics14111205 (PMC11171817; doi:10.3390/diagnostics14111205)
Supplement: Supplementary file 1 [file diagnostics-14-01205-s001.zip › diagnostics-3015218-supplementary.pdf]

Table S1. Significantly different radiomics features between early-stage and advanced cohorts (p-values <0.01).

Modality	Feature	P-value
CT (n = 34)	CT_HU_Min	0.001
	CT_HU_Q1	<0.001
	CT_DISC_HU_Min	0.002
	CT_DISC_HISTO_Skewness	<0.001
	CT_DISC_HISTO_Kurtosis	0.001
	CT_DISC_HISTO_Entropy_log10	<0.001
	CT_DISC_HISTO_Entropy_log2	<0.001
	CT_DISC_HISTO_Uniformity	<0.001
	CT_SHAPE_Volume (mL)	0.009
	CT_SHAPE_Volume (Voxel)	0.001
	CT_SHAPE_Sphericity	<0.001
	CT_SHAPE_Surface (mm^2)	0.001
	CT_GLCM_Homogeneity	<0.001
	CT_GLCM_Energy	0.001
	CT_GLCM_Contrast	<0.001
	CT_GLCM_Entropy_log10	<0.001
	CT_GLCM_Entropy_log2	<0.001
	CT_GLCM_Dissimilarity	<0.001
	CT_GLRM_SRE	<0.001
	CT_GLRLM_LRE	<0.001
	CT_GLRLM_SRLGE	0.009
	CT_GLRLM_SRHGE	0.002
	CT_GLRLM_LRLGE	0.002
	CT_GLRLM_LRHGE	<0.001
	CT_GLRLM_GLNU	<0.001
	CT_GLRLM_RLNU	0.007
	CT_GLRLM_RP	<0.001
	CT_NGLDM_Contrast	<0.001
	CT_NGLDM_Busyness	<0.001
	CT_GLZLM_LZE	0.001
	CT_GLZLM_LZLGE	0.001
	CT_GLZLM_LZHGE	0.001
	CT_GLZLM_GLNU	0.006
	CT_GLZLM_ZP	<0.001
PET_Whole (n = 30)	SUVmin	<0.001
	SUVmean	0.001
	SUV_Q1	<0.001
	SUV_Q2	<0.001
	SUV_Q3	0.002
	DISC_SUVmin	<0.001
	DISC_SUVmean	<0.001
	DISC_SUVmax	<0.001
	DISC_SUV_Q1	<0.001
	DISC_SUV_Q2	<0.001
	DISC_SUV_Q3	0.002
	DISC_SUV_Skewness	0.006
	DISC_HISTO_Skewness	0.003
	DISC_HISTO_Entropy_log10	0.004
	DISC_HISTO_Entropy_log2	0.004

	SHAPE_Sphericity	0.002
	SHAPE_Surface (mm^2)	0.001
	GLCM_Dissimilarity	0.008
	GLRLM_LGRE	<0.001
	GLRLM_HGRE	<0.001
	GLRLM_SRLGE	<0.001
	GLRLM_SRHGE	<0.001
	GLRLM_LRLGE	0.002
	GLZLM_SZE	0.001
	GLZLM_LGZE	0.001
	GLZLM_HGZE	<0.001
	GLZLM_SZLGE	0.004
	GLZLM_SZHGE	0.001
	GLZLM_GLNU	0.009
	GLZLM_ZP	0.004
**PET_40% (n = 21)**	DISC_SUVmean	0.006
	DISC_SUVmax	<0.001
	DISC_SUV_Q1	0.008
	DISC_SUV_Q2	0.008
	DISC_SUV_Q3	0.006
	DISC_TLG	0.003
	DISC_HISTO_Entropy_log10	0.007
	DISC_HISTO_Entropy_log2	0.007
	SHAPE_Volume (mL)	<0.001
	SHAPE_Volume (Voxel)	0.004
	SHAPE_Sphericity	<0.001
	SHAPE_Surface (mm^2)	<0.001
	GLCM_Entropy_log10	0.008
	GLCM_Entropy_log2	0.008
	GLRLM_LGRE	0.006
	GLRLM_SRLGE	0.005
	GLRLM_RLNU	<0.001
	GLZLM_LGZE	0.005
	GLZLM_SZLGE	0.009
	GLZLM_GLNU	0.001
	GLZLM_ZLNU	<0.001
**PET_70% (n = 13)**	DISC_SUVmin	0.005
	DISC_SUVmean	0.002
	DISC_SUVmax	<0.001
	DISC_SUV_Q1	0.004
	DISC_SUV_Q2	0.002
	DISC_SUV_Q3	0.001
	DISC_TLG	0.003
	SHAPE_Sphericity	<0.001
	SHAPE_Surface (mm^2)	0.005
	GLRLM_SRHGE	0.001
	GLZLM_SZHGE	0.009
	GLZLM_ZLNU	0.001
	GLZLM_ZP	0.007
**PET_Peak (n = 6)**	DISC_SUVmin	0.007
	DISC_SUVmean	0.004
	DISC_SUVmax	0.003
	DISC_SUV_Q1	0.006
	DISC_SUV_Q2	0.004
	DISC_SUV_Q3	0.004

Table S2. Significant features in the univariate Cox analysis (p-values <0.05).

Modality	Parameter	HR (95%CI)	p-value
Clinical (n = 6)	Having Advanced Disease	2.606 (1.921-3.535)	<0.001
	Age (continuous)	1.017 (1.004-1.031)	0.012
	Age >70 (categorical)	1.545 (1.136-2.102)	0.006
	ECOG ≥2	3.403 (2.286-5.066)	<0.001
	Sarcopenia Score	0.973 (0.957-0.990)	0.002
	Being Sarcopenic	1.871 (1.385-2.526)	<0.001
	Having SCC/Undifferentiated Pathology	1.580 (1.102-2.265)	0.013
CT (n = 4)	CT_SHAPE_Volume (mL)	1.006 (1.002-1.010)	0.002
	CT_SHAPE_Sphericity	0.064 (0.015-0.270)	<0.001
	CT_NGLDM_Contrast	0.006 (0.000-0.389)	0.016
	CT_GLZLM_GLNU	1.001 (1.000-1.002)	0.009
PET_Whole (n = 9)	SHAPE_Volume (mL)	1.007 (1.004-1.010)	<0.001
	SHAPE_Sphericity	0.089 (0.024-0.338)	<0.001
	SHAPE_Compacity	1.162 (1.014-1.333)	0.031
	GLRLM_GLNU	1.002 (1.001-1.004)	0.009
	NGLDM_Coarseness	0.001 (0.001-0.157)	0.025
	NGLDM_Busyness	1.391 (1.048-1.847)	0.022
	GLZLM_SZE	3.025 (0.979-9.544)	0.049
	GLZLM_GLNU	1.010 (1.003-1.016)	0.002
	GLZLM_ZLNU	1.001 (1.001-1.002)	<0.001
PET_40% (n = 9)	CONVENTIONAL_TLG	1.001 (1.000-1.001)	0.001
	SHAPE_Volume (mL)	1.009 (1.004-1.014)	0.001
	SHAPE_Sphericity	0.105 (0.028-0.390)	0.001
	GLRLM_LRE	1.054 (1.019-1.089)	0.002
	GLRLM_SRLGE	0.001 (0.001-0.082)	0.044
	GLRLM_GLNU	1.002 (1.000-1.003)	0.011
	NGLDM_Coarseness	0.001 (0.001-0.041)	0.015
	GLZLM_GLNU	1.011 (1.002-1.020)	0.013
	GLZLM_ZLNU	1.002 (1.001-1.004)	0.001
PET_70% (n = 4)	CONVENTIONAL_TLG	1.003 (1.002-1.004)	<0.001
	DISCRETIZED_TLG	1.001 (1.000-1.002)	<0.001
	SHAPE_Volume (mL)	1.042 (1.015-1.069)	0.002
	SHAPE_Volume (Voxel)	1.002 (1.001-1.003)	<0.001

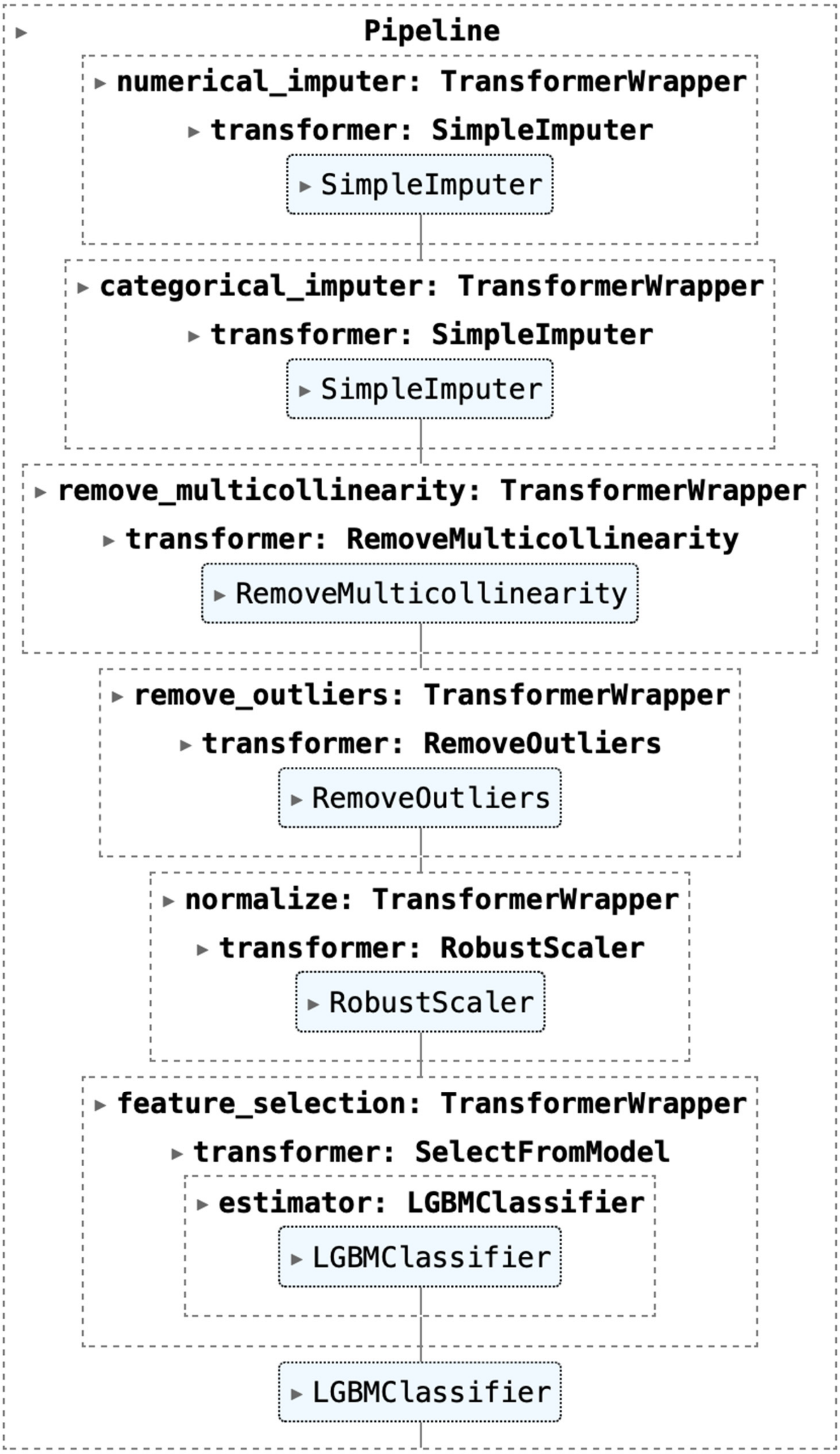

Figure S1. Light gradient boosting machine (LGBM) pipeline.

Details of the important features in each model building combination (LGBM Classifier)

Baseline Clinical Model (Age, Sex, Race, BMI, ECOG, Histology, Grade)

Accuracy: 0.64, AUC: 0.70, Recall: 0.60, Precision: 0.68, F1: 0.63

CT-Derived Radiomics Model

Important features to include:

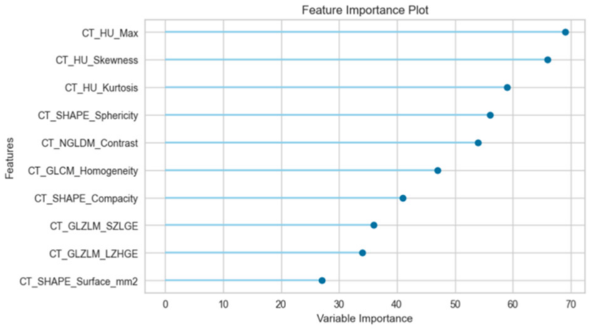

Accuracy: 0.72, AUC: 0.78, Recall: 0.77, Precision: 0.73, F1: 0.74

PET-Derived Radiomics Features

Important features to include:

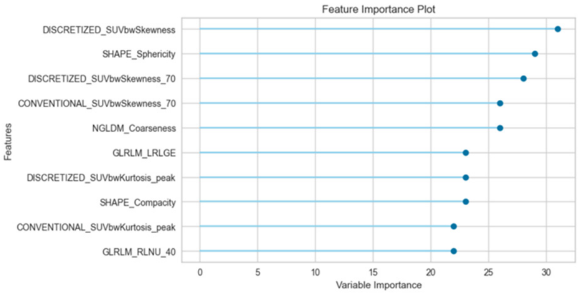

Accuracy: 0.51, AUC: 0.54, Recall: 0.57, Precision: 0.54, F1: 0.55

PET/CT-Derived Radiomics Features

Important features to include:

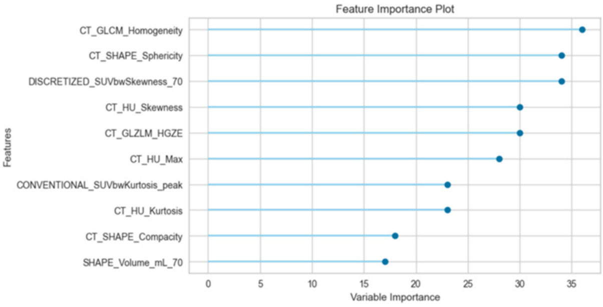

Accuracy: 0.67, AUC: 0.78, Recall: 0.71, Precision: 0.69, F1: 0.34

CT-Derived Radiomics Feature + Clinical Data

Important features to include:

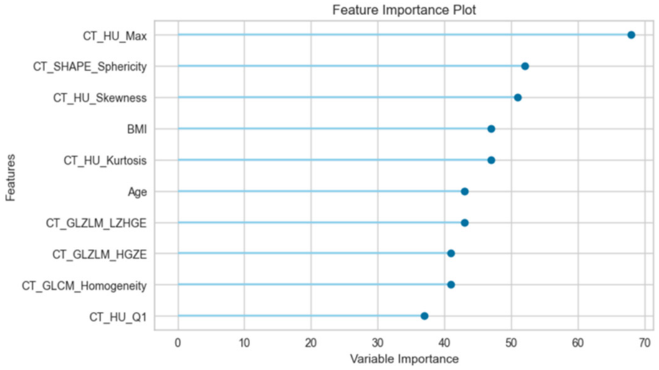

Accuracy: 0.76, AUC: 0.84, Recall: 0.78, Precision: 0.77, F1: 0.77

CT-Derived Features + Sarcopenia Score

Important features to include:

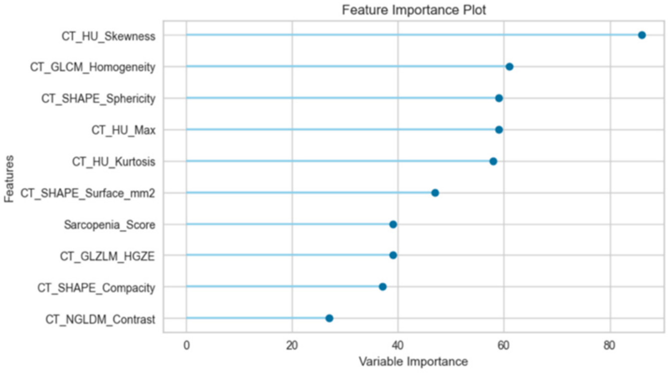

Accuracy: 0.68, AUC: 0.77, Recall: 0.72, Precision: 0.69, F1: 0.70

CT-Derived Features + Clinical Data + Sarcopenia Score

Important features to include:

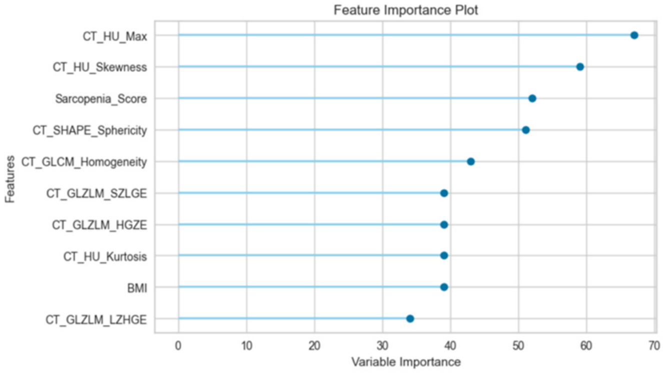

Accuracy: 0.77, AUC: 0.83, Recall: 0.79, Precision: 0.79, F1: 0.78

PET-Derived Radiomics Feature + Clinical Data

Important features to include:

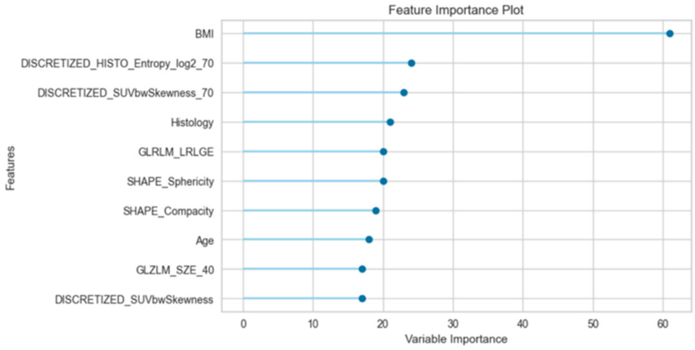

Accuracy: 0.69, AUC: 0.77, Recall: 0.67, Precision: 0.72, F1: 0.68

PET-Derived Radiomics Feature + Sarcopenia Score

Important features to include:

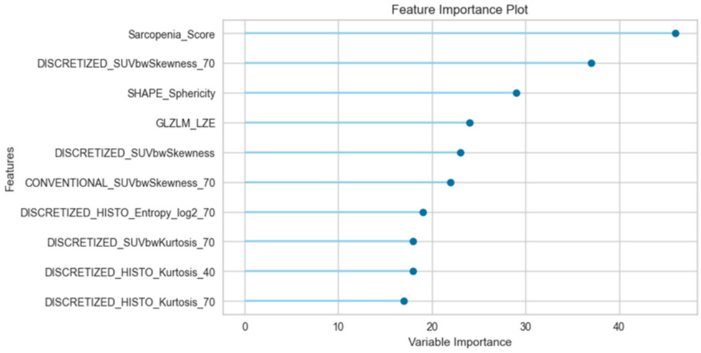

Accuracy: 0.68, AUC: 0.69, Recall: 0.72, Precision: 0.69, F1: 0.70

PET-Derived Radiomics Feature + Clinical Data + Sarcopenia Score

Important features to include:

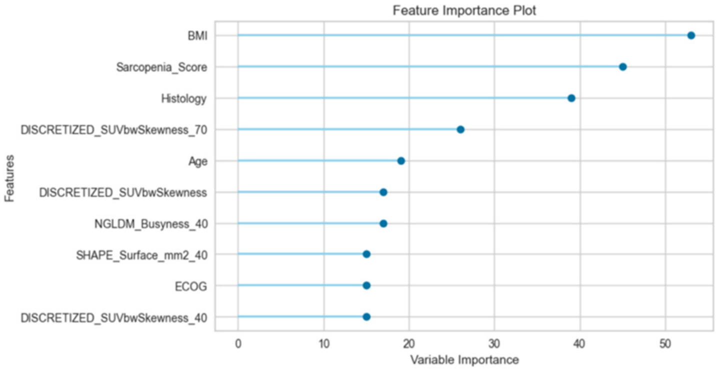

Accuracy: 0.74, AUC: 0.80, Recall: 0.74, Precision: 0.76, F1: 0.75

PET/CT-Derived Radiomics Feature + Clinical Data

Important features to include:

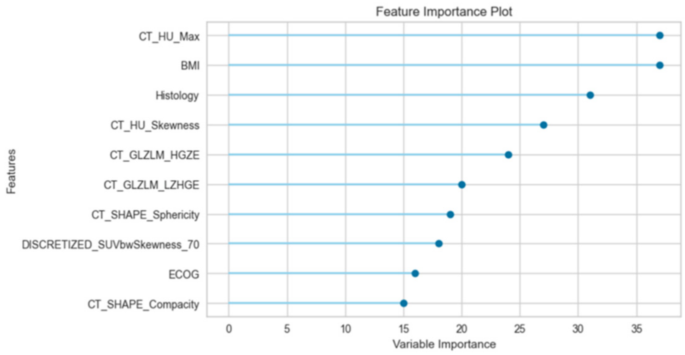

Accuracy: 0.76, AUC: 0.85, Recall: 0.80, Precision: 0.77, F1: 0.78

PET/CT-Derived Radiomics Feature + Sarcopenia Score

Important features to include:

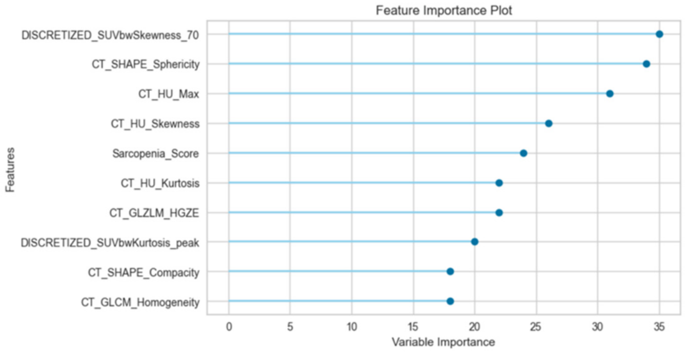

Accuracy: 0.71, AUC: 0.78, Recall: 0.76, Precision: 0.72, F1: 0.73

PET/CT-Derived Radiomics Feature + Clinical Data + Sarcopenia Score

Important features to include:

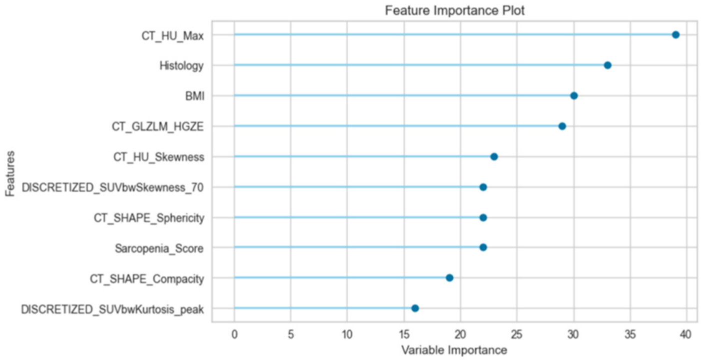

Accuracy: 0.79, AUC: 0.85, Recall: 0.80, Precision: 0.80, F1: 0.80

Important features in each model building combination (Random Forest classifier)

Baseline Clinical Model (Age, Sex, Race, BMI, ECOG, Histology, Grade)

Accuracy: 0.63, AUC: 0.69, Recall: 0.63, Precision: 0.66, F1: 0.64

CT-Derived Radiomics Model

Important features to include:

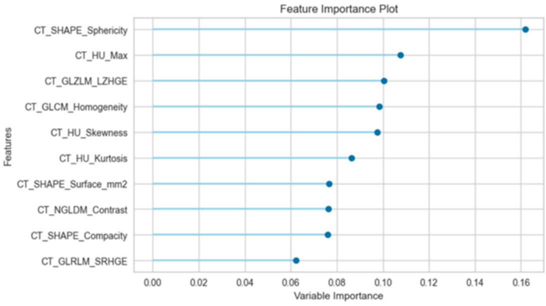

Accuracy: 0.72, AUC: 0.79, Recall: 0.78, Precision: 0.72, F1: 0.74

PET-Derived Radiomics Features

Important features to include:

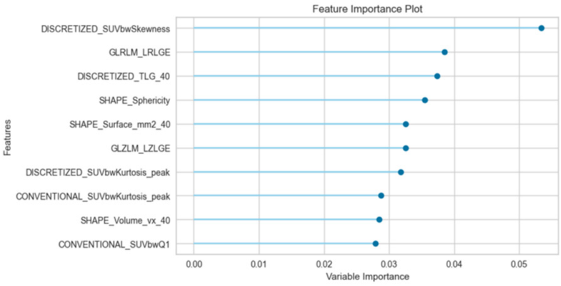

Accuracy: 0.57, AUC: 0.62, Recall: 0.64, Precision: 0.59, F1: 0.61

PET/CT-Derived Radiomics Features

Important features to include:

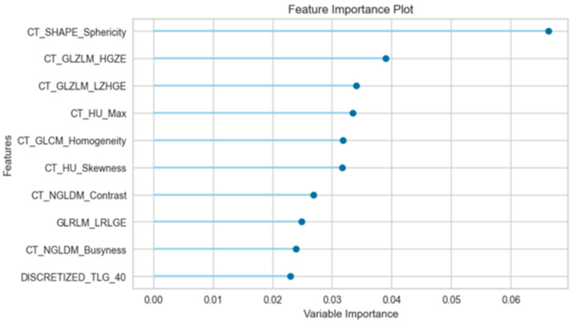

Accuracy: 0.72, AUC: 0.74, Recall: 0.76, Precision: 0.73, F1: 0.74

CT-Derived Radiomics Feature + Clinical Data

Important features to include:

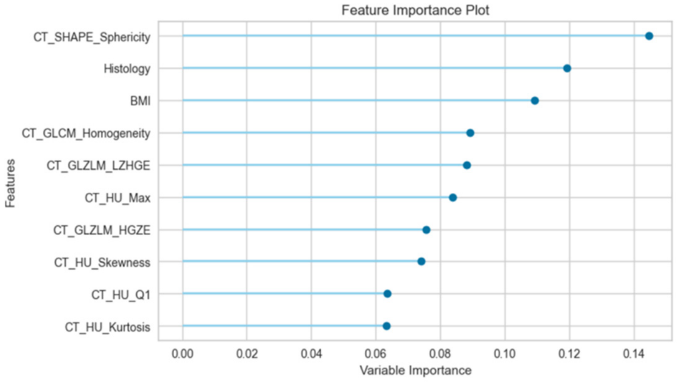

Accuracy: 0.75, AUC: 0.85, Recall: 0.76, Precision: 0.79, F1: 0.76

CT-Derived Features + Sarcopenia Score

Important features to include:

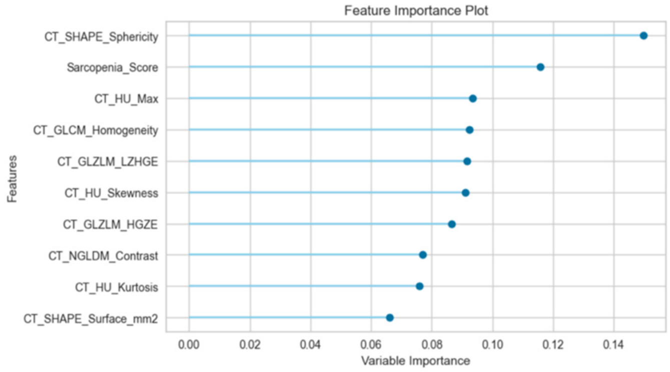

Accuracy: 0.71, AUC: 0.79, Recall: 0.73, Precision: 0.72, F1: 0.73

CT-Derived Features + Clinical Data + Sarcopenia Score

Important features to include:

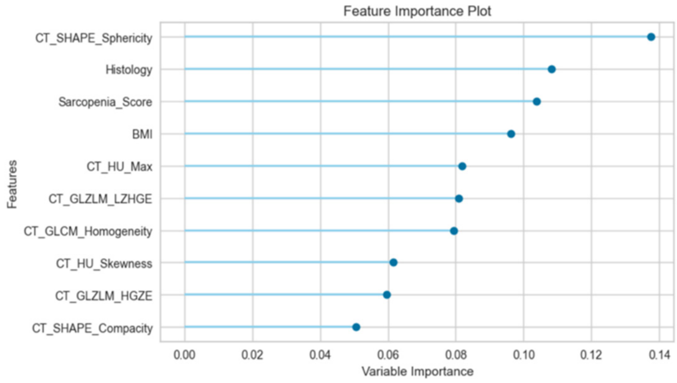

Accuracy: 0.74, AUC: 0.82, Recall: 0.79, Precision: 0.75, F1: 0.76

PET-Derived Radiomics Feature + Clinical Data

Important features to include:

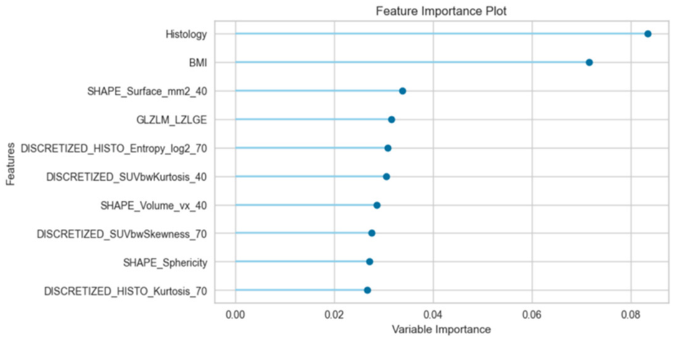

Accuracy: 0.71, AUC: 0.77, Recall: 0.74, Precision: 0.71, F1: 0.72

PET-Derived Radiomics Feature + Sarcopenia Score

Important features to include:

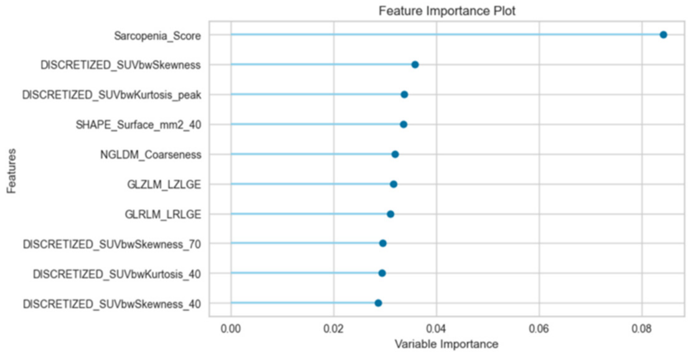

Accuracy: 0.65, AUC: 0.67, Recall: 0.72, Precision: 0.65, F1: 0.68

PET-Derived Radiomics Feature + Clinical Data + Sarcopenia Score

Important features to include:

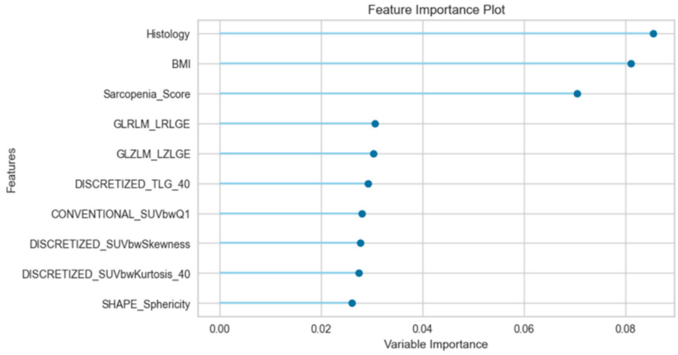

Accuracy: 0.72, AUC: 0.80, Recall: 0.73, Precision: 0.77, F1: 0.74

PET/CT-Derived Radiomics Feature + Clinical Data

Important features to include:

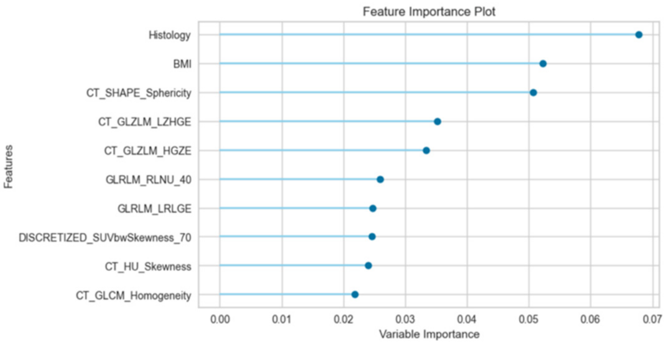

Accuracy: 0.72, AUC: 0.80, Recall: 0.73, Precision: 0.77, F1: 0.74

PET/CT-Derived Radiomics Feature + Sarcopenia Score

Important features to include:

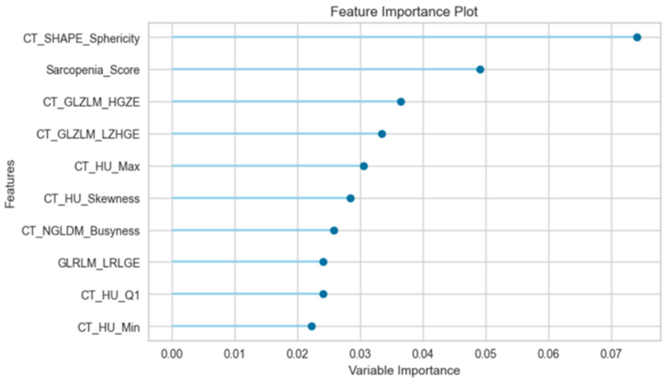

Accuracy: 0.71, AUC: 0.75, Recall: 0.73, Precision: 0.73, F1: 0.72

PET/CT-Derived Radiomics Feature + Clinical Data + Sarcopenia Score

Important features to include:

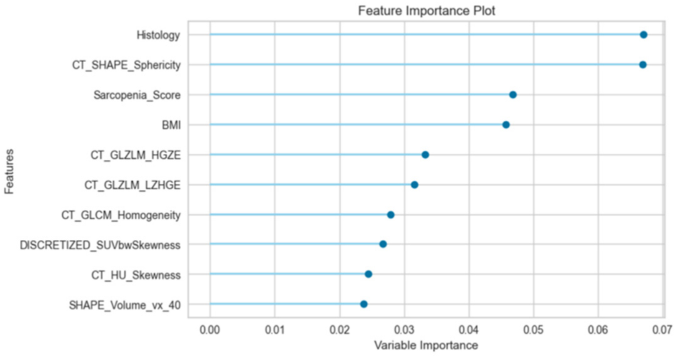

Accuracy: 0.76, AUC: 0.82, Recall: 0.79, Precision: 0.77, F1: 0.77
